# Supplementary material for: BmCDK5 Affects Cell Proliferation and Cytoskeleton Morphology by Interacting with BmCNN in Bombyx mori
Source: Insects. 2022 Jul 6;13(7):609. doi: 10.3390/insects13070609 (PMC9323621; doi:10.3390/insects13070609)
Supplement: Supplementary file 1 [file insects-13-00609-s001.zip › insects-1776588-supplementary.pdf]

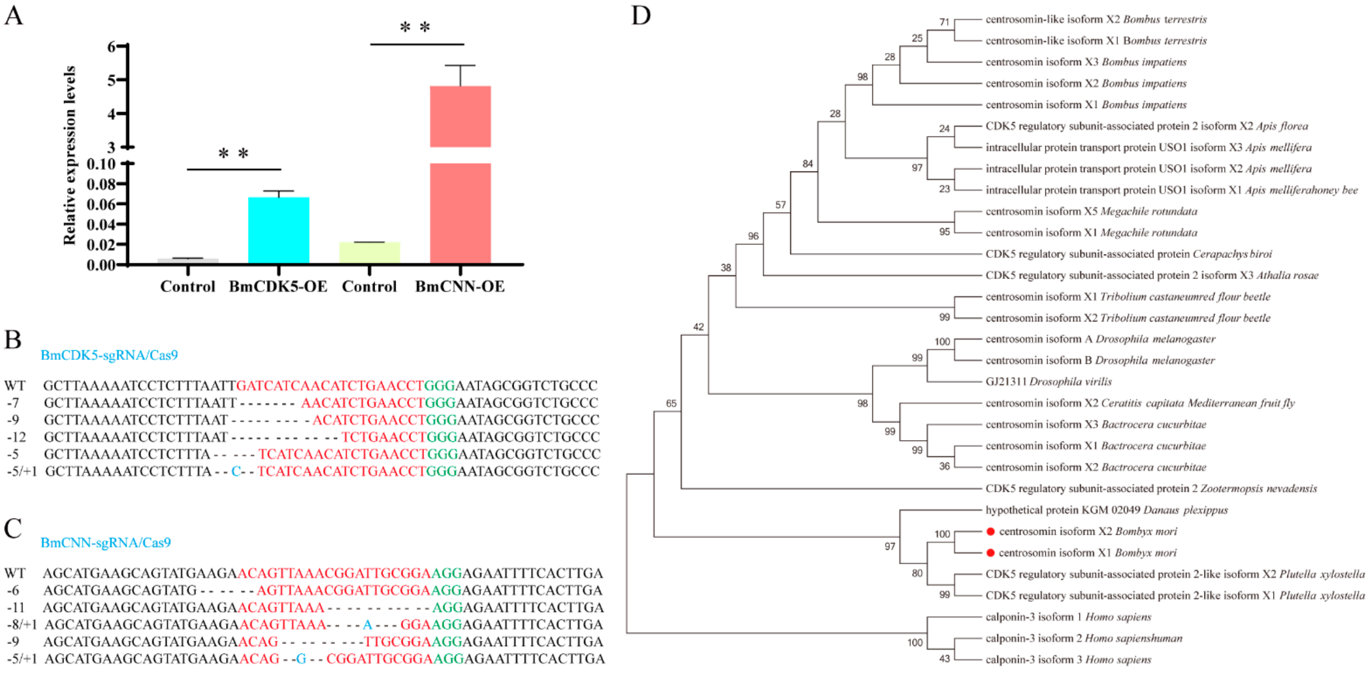

Figure. S1. (A) The level of transcription of *BmCDK5 and BmCNN* in *BmCDK5* overexpressing BmNS cells and empty vector control cells was determined using qRT-PCR. The level of transcription of *BmCNN* in *BmCNN* overexpressing BmNS cells and empty vector control cells was determined using qRT-PCR (* $p < 0.05$, ** $p < 0.01$). (B-C) DNA sequencing analysis of the CRISPR/Cas9 editing target genes (BmCDK5 and BmCNN). The red sequence is the target sequence. WT is the unknocked sample, as a reference. (D) Phylogenetic tree of CNN.

Table S1. The RT-qPCR primer sequences.

**Primers**	**Sequences** (5’-3’)
QBmCDK5/F	GATGTATTGCACAGCGAAAAGA
QBmCDK5/R	CTTCAGCGGAATAGCACTTTAC
QBmCNN/F	GCACAACGTTCGATTACAAGTA
QBmCNN/R	TTTCATCGAAGCGCTATTGTTC
BmCDK5/F	CGCGGATCCATGGATTACAAGGATGACGACGATAAGATGCAAAAATAT
BmCDK5/R	CCGGAATTCTTAACATCGATCATTCTTGACTG

BmCNN/F	CCGGAATTCATGTACCCATACGATGTTCCAGATTACGCTATGGCCACATTACCTAGAAC
BmCNN/R	CCGCTCGAGTTACATATTACTATGAGTTTCG
sgBmCDK5/F	AAGTGATCATCAACATCTGAACCT
sgBmCDK5/R	AAACAGGTTCAGATGTTGATGATC
sgBmCNN/F	AAGTACAGTTAAACGGATTGCGGA
sgBmCNN/R	AAACTCCGCAATCCGTTTAACTGT
Qsw22934/F	AACACCCCGTCCTGCTCACTG
Qsw22934/R	GGGCGAGACGTGTGATTTCCT
M13/F	GTTTTCCCAGTCACGAC
M13/R	CAGGAAACAGCTATGAC
U6/F	GGAAGATCTTCCAGGTTATGTAGTACACATTGT
U6/R	GGAAGATCTTCCAAAAAAAGCACCGACTCG
T-BmCDK5/F	CGCGGATCCATGCAAAAATATGAG
T-BmCDK5/R	GGAGATTTATTATGATGAACTCACC
T-BmCNN/F	TACTGGTGGTAGTACTTAGA
T-BmCNN/R	ATATCGATTTTATCAAGCAC
